# Characterization and quantification of flavonoids and saponins in adzuki bean (*Vigna angularis* L.) by HPLC–DAD–ESI–MS^n^ analysis

**DOI:** 10.1186/s13065-017-0317-x

**Published:** 2017-09-22

**Authors:** Rui Liu, Zongwei Cai, Baojun Xu

**Affiliations:** 1 Food Science and Technology Program, Beijing Normal University-Hong Kong Baptist University United International College, 28, Jinfeng Road, Tangjiawan, Zhuhai, 519085 Guangdong China; 2 Department of Chemistry, Hong Kong Baptist University, Kowloon, Hong Kong China

**Keywords:** Adzuki bean, Flavonoids, Saponins, ESI–MS^n^, HPLC

## Abstract

**Background:**

Bioactive activities of adzuki bean have been widely reported, however, the phytochemical information of adzuki bean is incomplete. The aim of this study was to characterize and quantify flavonoids and saponins in adzuki bean. High performance liquid chromatography with diode array detection and electro spray ionization-tandem multi-stage mass spectrometry (HPLC–DAD–ESI–MS^n^) were applied to do qualitative and quantitative analyses.

**Results:**

A total of 15 compounds from adzuki bean were identified by HPLC–DAD–ESI–MS^n^. Among 15 compounds identified, four flavonoids (catechin, vitexin-*4″*-*O*-glucoside, quercetin-*3*-*O*-glucoside, and quercetin-*3*-*O*-rutinoside) and six saponins (azukisaponin I, II, III, IV, V, and VI) in adzuki bean were further quantified by external calibration method using HPLC–MS with the program of time segment and extract ion chromatogram (EIC) analysis.

**Conclusions:**

Current qualitative and quantitative method based on HPLC and MS technique provides a scientific basis for in vitro and in vivo pharmacological study in the future.

**Graphical abstract:**
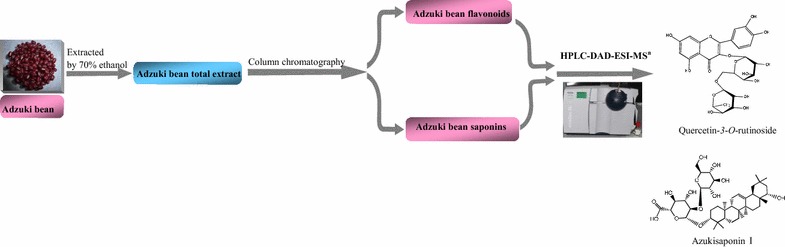
Isolation and characterization of flavonoids and saponins from adzuki bean.

## Introduction

Adzuki bean is mainly produced and consumed in China and several other countries in East Asia. It has been used as a diuretic, antidote, and remedy for dropsy and beriberi in traditional Chinese medicine and also used as food for thousands of years. Extensive bioactivities of adzuki bean, such as anti-tumor [1, 2], anti-diabetes, [3, 4], antioxidant [5–8], and hepatoprotective effect [9] have been reported. These bioactivities are contributed by chemical constituents in beans, mainly including flavonoids and saponins. The previous articles showed that adzuki bean contained flavonoids such as (+) epicatechin, (+) catechin, quercetin, vitexin or their derivatives; [6, 10, 11] and saponins, such as azukisaponin I, II, III, IV, V, and VI [12] and AZ I [13], II, III, and IV [14]. The information, especially saponin information on adzuki bean, is incomplete, the name and structure of “AZ” and azukisaponin are confused. Moreover, there are limited articles in recent years to systematically and comprehensively investigate flavonoids and saponins of adzuki bean. Therefore, the present study aimed to establish a method to separate individual flavonoids and saponins from adzuki bean, characterize their chemical structures by HPLC–DAD–ESI–MS^n^, and further quantify them by HPLC–MS.

## Materials and methods

### Materials

Adzuki beans (*Vigna angularis* L.) were purchased from local market in Changchun, Jilin Province, and identified by Prof. Jinming Mu of Faculty of Agronomy in Jilin Agricultural University.

### Chemicals and reagents

Chromatographic grade acetonitrile and methanol were purchased from Merck (Darmstadt, USA). (+) Catechin, (+) epicatechin, quercetin-*3*-*O*-rutinoside, quercetin-*3*-*O*-glucoside and vitexin-*4″*-*O*-glucoside were purchased from Sigma (St. Louis, MO, USA). Saponin standards of azukisaponin I, II, III, IV, V, and VI were isolated in our lab. Other chemicals, such as ethanol, methanol, and acetone were of analytical grade. Macro porous resins AB-8 were supplied by Nankai University. Polyamide resin was purchased from Sinopharm Chemical Reagent Co., Ltd. (Beijing, China).

### Sample preparation of flavonoids and saponins from adzuki bean

**Fig. 1:**
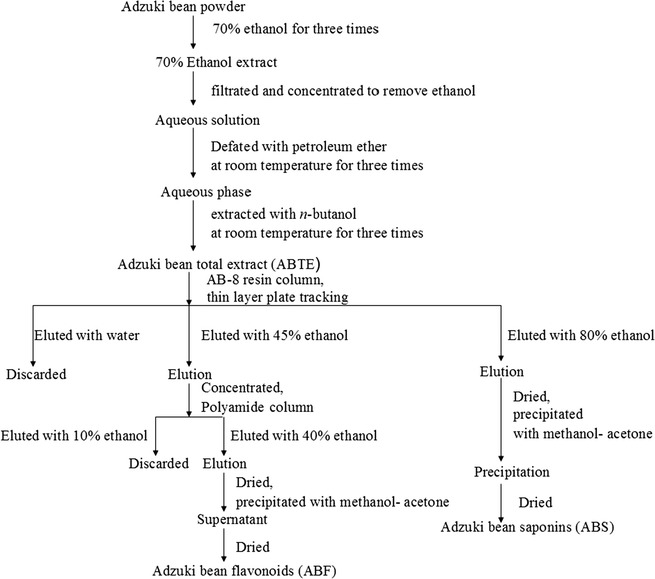
Extraction and isolation scheme of total extract, flavonoids and saponins from adzuki bean

The flavonoids and saponins of adzuki bean were prepared according to the previous articles [15–18]. Extraction and isolation scheme of total extract, flavonoids and saponins from adzuki bean was shown in Fig. 1. Briefly, adzuki bean was ground, 14 kg of the powder was then extracted with 140 L of 70% ethanol for three times. The combined extracts were filtrated and concentrated to remove ethanol. The remaining aqueous solution was extracted with 14 L of petroleum ether at room temperature for three times. The aqueous phase was then extracted with 14 L of *n*-butanol at room temperature three times. The *n*-butanol layer was evaporated under vacuum to obtain 158.6 g of extract which was defined as adzuki bean total extract (ABTE). Flavonoids of adzuki bean were collected from the 45% ethanol elution fraction from AB-8 resin column after eluting with water. The collected crude flavonoids of adzuki bean were subjected to the second column with polyamide according to the literature [19–21], and the flavonoids were further obtained in 40% ethanol fraction from polyamide column after eluting with 10% ethanol. Finally, the enriched adzuki bean flavonoids (ABF) were obtained from the supernatant after precipitating with methanol–acetone. The enriched saponin of adzuki bean was collected in the 80% ethanol fraction from AB-8 resin column after eluting with 45% ethanol. With precipitation method, adzuki bean saponins (ABS) were further purified using precipitation method by adding methanol–acetone.

### High performance liquid chromatography analysis

The chemical constituents of adzuki bean total extract (ABTE), adzuki bean flavonoids (ABF) and adzuki bean saponins (ABS) were identified by liquid chromatography-ion trap mass spectrometry. HPLC analysis was performed on an Agilent 1100 series HPLC system equipped with degasser, binary pump, diode array detector and auto-sampler (San Francisco, USA). The separation was performed on a Phenomenex C_8_ column (150 × 2.0 mm, 5 μm). Gradient elution was performed using water containing 10 mM ammonium acetate (A) and acetonitrile (B). Initial conditions were 10% B for 10 min, changed to 15% B at 30 min and 25% B at 45 min, and then 35% B at 55 min, 45% B at 60 min and 55% B at 70 min. Flow rate was set at 0.2 mL/min, and UV absorption was measured at wavelength of 205 nm and 262 nm for saponins and flavonoids, respectively. The sample injection volume was 10 μL.

### Electro spray ionization-tandem multi-stage mass spectrometry (ESI–MS^n^) analysis

ESI–MS analysis was carried out on an Esquire 4000 ion trap mass spectrometer (Bruker–Daltonics, Bremen, Germany) with an electrospray ionization (ESI) interface. The instrument was operated at an ionization voltage of +4000 V and source temperature of 300 °C. Nitrogen was used as nebulizer gas at 30 psi and drying gas at a flow rate of 9 L/min. Collision energy was optimized for each compound. Three time segments were used in mass spectrometric acquisition in order to optimize the instrumental parameters for each compound to increase the peak intensity. The full scan of ions ranging from *m/z* 100 to *m/z* 1200 in the negative ion mode was used. Retention times and MS chromatograms of all flavonoids and saponins were confirmed by authentic standards, respectively. The HPLC chromatograms and total ion chromatograms (TIC) were obtained using the above method.

## Results and discussion

### Optimization of sample preparation

Flavonoids distribute in nature widely, especially in a large number of biologically active natural products. Most flavonoids exhibit two major maximum UV absorption wavelengths, namely the range of 240–285 nm and the range of 300–400 nm. While most saponins exhibit no ultraviolet absorption. The current results revealed the presence of flavonoids mainly in 45% ethanol fractions and the presence of saponins mainly in 80% ethanol fractions by AB-8 resin column. It was reported that AB-8 resin was good at separating chemical constituent according to the polarity [22]. After that, polyamide column was employed to further purify the flavonoids. 40% Ethanol eluent from polyamide column was obtained, and the fraction was rich in flavonoids. Precipitation with methanol–acetone was applied to further separate flavonoids and saponins from adzuki bean. Flavonoids existed in the supernatant fraction, while saponins presented in the precipitate fraction. Finally, adzuki bean total extract (ABTE), adzuki bean flavonoids (ABF) and adzuki bean saponins (ABS) (Fig. 1) were obtained and utilized for HPLC–DAD–ESI–MS^n^ analysis.

### Optimization of HPLC–DAD–ESI–MS^n^ conditions

**Fig. 2:**
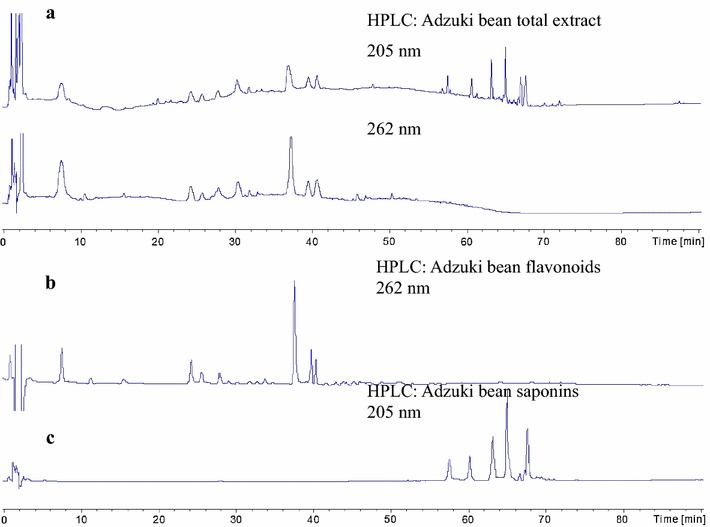
HPLC–DAD chromatograms of adzuki bean extracts. **a** HPLC–DAD chromatogram of adzuki bean total extract at 205 and 262 nm, respectively; **b** HPLC–DAD chromatogram of adzuki bean flavonoids at 262 nm; **c** HPLC–DAD chromatogram of adzuki bean saponins at 205 nm

A binary mobile phase of water/acetic acid (98:2, v/v) (solvent A) and water/acetonitrile/acetic acid (78:20:2, v/v/v) (solvent B) with gradient program to separate flavonoids such as quercetin derivatives, cinnamic acid derivatives and kaempferol derivatives were applied previously [10]. A mixture of solvent A (HPLC water containing 0.05% TFA) and solvent B (acetonitrile: methanol: TFA = 30:10:0.05) was also used to separate phenolics including flavonoids and their derivatives in another report [23]. In current article, several aqueous mobile phases, consisting of methanol, water or acetonitrile and water (with or without adjusting pH value), or different buffers (such as ammonium acetate, ammonium formate and formic acid), with altered flow rates, and different gradient compositions, were used to optimize HPLC chromatographic conditions. The results showed that the mobile phase of water containing 10 mM ammonium acetate combined with mobile phase B containing acetonitrile were feasible for HPLC–MS system. The flow rate was set at 0.2 mL/min, the gradient eluting conditions were 10% B for 10 min, changed to 15% B at 30 min, 25% B at 45 min, 35% B at 55 min, 45% B at 60 min and 55% B at 70 min. Such conditions exhibited good separation for both flavonoids and saponins (Fig. 2). Previously, Alltima C_18_ column [23], and Phenomenex Luna C_18_ column [10] were respectively used to separate flavonoids such as catechin, vitexin, and quercetin. In current article, different chromatographic columns such as C_18_ column, C_8_ column, purchased from different companies (such as Agilent, Waters, Phenomenex, Shimadzu) were attempted, and finally the Phenomenex C_8_ column (150 × 2.0 mm, 5 μm) were selected. As regards to the selection of the wavelength, 205 nm was employed to detect oleanene-glucuronides in commercial edible beans [24], while 230 nm [25], 280 nm [23], and 320 nm [10] were used to monitor phenolics including flavonoids and their derivatives. Therefore, the current working wavelength at 205 nm for detecting saponins and the wavelength of 262 nm for detecting flavonoids were set according to the preliminary experiments. The HPLC chromatograms of adzuki bean total extract (205 and 262 nm) were presented in Fig. 2a. The peaks of HPLC chromatogram detected at 262 nm disappeared after 50 min, while the peaks of HPLC chromatogram detected under 205 nm showed up after 50 min. Figure 2b showed the chromatogram (262 nm) of adzuki bean flavonoids. Figure 2c exhibited the chromatogram (205 nm) of adzuki bean saponins.

**Fig. 3:**
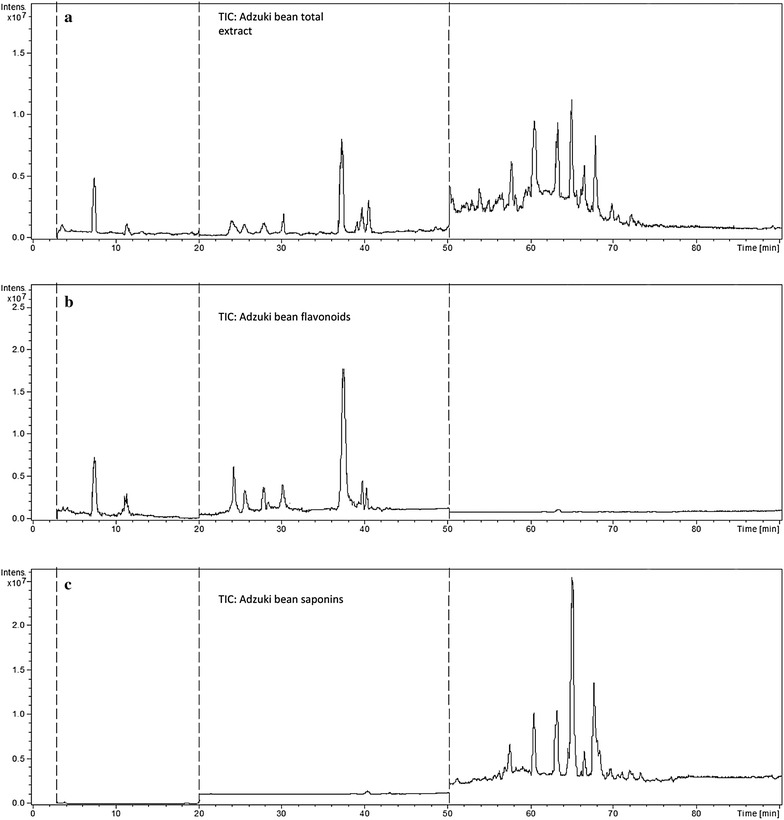
HPLC–ESI–MS total ion chromatograms of adzuki bean extracts. **a** HPLC–ESI–MS total ion chromatogram of adzuki bean total extract; **b** HPLC–ESI–MS total ion chromatogram of adzuki bean flavonoids; **c** HPLC–ESI–MS total ion chromatogram of adzuki bean saponins

Subsequently, electro spray ionization (ESI) conditions for detecting flavonoids and saponins were optimized. A direct infusion experiment was firstly employed in negative ion detection mode. Under the optimized MS conditions, the *m/z* 289 precursor ion was identified as catechin. The *m/z* 609 precursor ion, which produced the *m/z* 463, *m/z* 301 daughter ions, was identified as quercetin-*3*-*O*-rutinoside. The *m/z* 463 precursor ion, which produced the *m/z* 301 daughter ion, was identified as quercetin-*3*-*O*-glucoside. The *m/z* 431 precursor ion was identified as vitexin. The *m/z* 593 precursor ion, which produced the *m/z* 431 daughter ion, was identified as vitexin 4″-*O*-glucoside. In order to detect the chemical constituents effectively and simultaneously, the program of time segments with different MS conditions were used in this article. Figure 3 showed HPLC–ESI–MS total ion chromatograms of adzuki bean samples. The authors focused on 15 major peaks as marked in Fig. 3a for further structural analysis. Similar to the saponins standards, the *m/z* 779, *m/z* 795, *m/z* 809, *m/z* 971, *m/z* 941, and *m/z* 1133 precursor ions were for azukisaponin I, II, III, IV, V, and VI, respectively. The detailed MS^n^ information of azukisaponins was discussed in the following identification analysis.

### Analysis of flavonoids in adzuki bean by HPLC–ESI–MS^n^

**Fig. 4:**
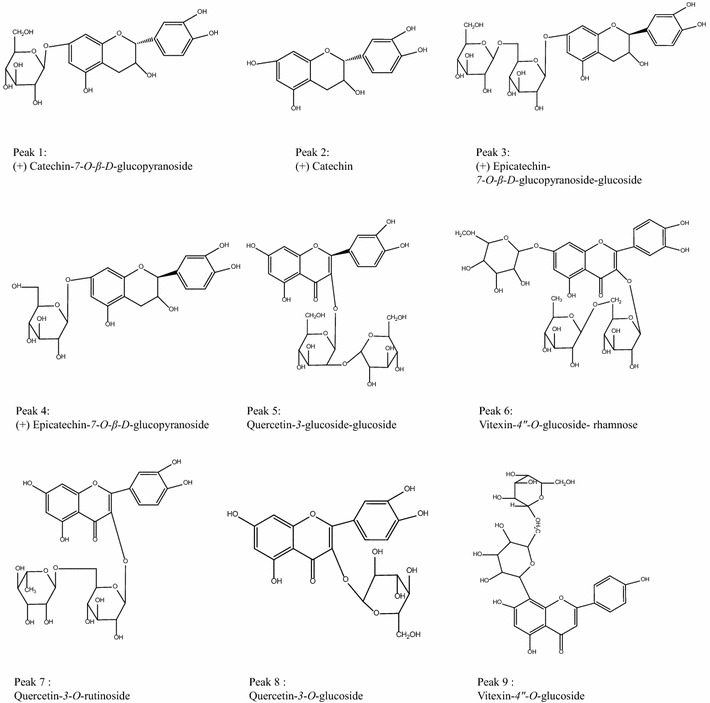
Chemical structures of flavonoids in adzuki bean identified by HPLC–ESI–MS^n^

A total of 15 major peaks in HPLC–ESI–MS total ion chromatograms of adzuki bean total extract were marked in Fig. 3a. The information of the retention times, *m/z* for the [M−H]^−^ ions and the collision induced dissociation (CID) fragments of peaks were listed in Table 1. Peaks 1–9 were identified as flavonoids by HPLC–DAD–ESI–MS^n^ by comparing the retention times and ESI–MS^n^ spectra with those of authentic standards, and the chemical structures of these flavonoids were shown in Fig. 4.

**Table 1:** HPLC–ESI–MS^n^ data of identified flavonoids and saponins in adzuki bean

Peak no.	Retention time (min)	–MS [M−H]^−^ (*m/z*)	Daughter ion of MS^2^ (*m/z*)	Daughter ion of MS^3^ (*m/z*)	Identity
1	7.5	451	289		(+) Catechin-*7*-*O*-*β*-*d*-glucopyranoside
2	11.3	289			(+) Catechin
3	24.1	613	451	289	(+) Epicatechin-*7*-*O*-*β*-*d*-glucopyranoside-glucoside
4	25.7	451	289		(+) Epicatechin-*7*-*O*-*β*-*d*-glucopyranoside
5	27.9	625	493 463	463, 301 301	Quercetin-*3*-glucoside–glucoside
6	30.1	739	593 431	431, 413	Vitexin-*4″*-*O*-glucoside-rhamnose
7	37.5	609	463	301	Quercetin-*3*-*O*-rutinoside
8	39.6	463	301		Quercetin-*3*-*O*-glucoside
9	40.5	593	431	413	Vitexin-*4″*-*O*-glucoside
10	57.7	971	809 647 629 485	647, 485 485	Azukisaponin IV
11	60.4	1133	809 629 471	629, 471	Azukisaponin VI
12	63.2	941	795 633 457	633, 615, 457 615, 457	Azukisaponin V
13	64.9	795	633 457	615, 457	Azukisaponin II
14	66.4	779	617 599 441	599, 441	Azukisaponin I
15	67.7	809	647 471	471	Azukisaponin III

**Fig. 5:**
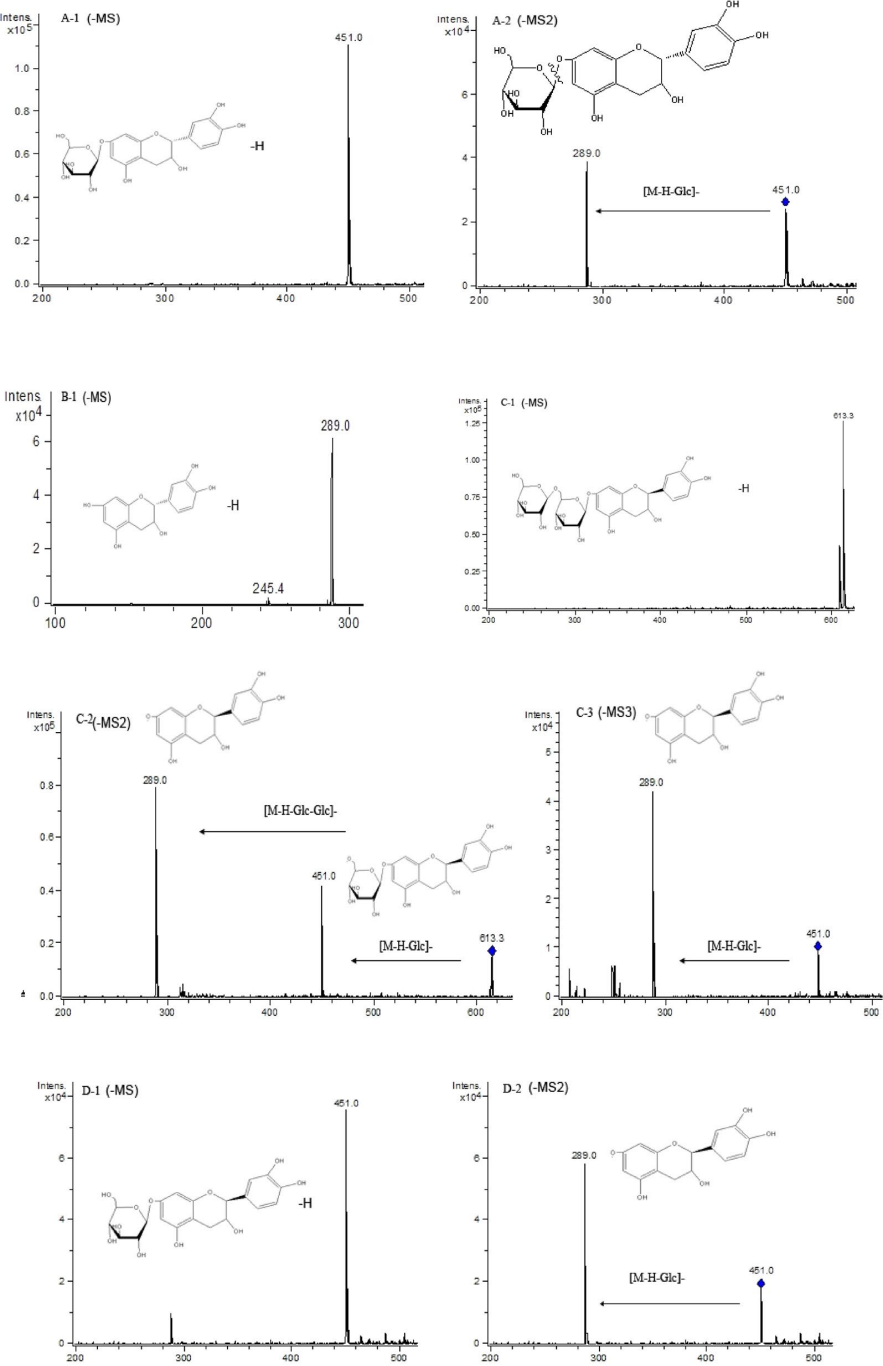
ESI (−) MS, MS^2^, and MS^3^ spectra of identified flavonoids in adzuki bean. Peak 1 (**A**): **A-1** MS spectrum of peak 1 ([M−H]^−^), **A-2** MS^2^ spectrum of the ion at *m/z* 451. Peak 2 (**B**): **B-1** MS spectrum of peak 2 ([M−H]^−^). Peak 3 (**C**): **C-1** MS spectrum of peak 3 ([M−H]^−^), **C-2** MS^2^ spectrum of the ion at *m/z* 613, **C-3** MS^3^ spectrum of the ion at *m/z* 451. Peak 4 (**D**): **D-1** MS spectrum of peak 4 ([M−H]^−^), **D-2** MS^2^ spectrum of the ion at *m/z* 451

For peak 1, the retention time was 7.5 min, and the *m/z* 451 precursor ion was presented in Fig. 5A-1. As shown in Fig. 5A-2, the *m/z* 289 daughter ion was the main fragment ion of the parent ion at *m/z* 451. Moreover, the *m/z* 289 ion was the precursor ion of (+) catechin. Therefore, peak 1 was identified as (+) catechin-*7*-*O*-*β*-*d*-glucopyranoside according to the above information and the previous article [26]. The retention time of peak 2 was 11.3 min, and peak 2 was identified to be (+) catechin comparing the retention time and the ESI (−)-MS spectra with the authentic standard (+) catechin. The *m/z* 289 precursor ion for peak 2 was presented in Fig. 5B. Peak 3 was speculated to be (+) epicatechin-*7*-*O*-*β*-*d*-glucopyranoside-glucoside with the retention time 24.1 min and *m/z* 613 (Fig. 5C-1) as the precursor ion [M−H]^−^. As shown in Fig. 5C-2, the *m/z* 451 and *m/z* 289 daughter ions were the main fragment ions of the parent ion at *m/z* 613.

**Fig. 6:**
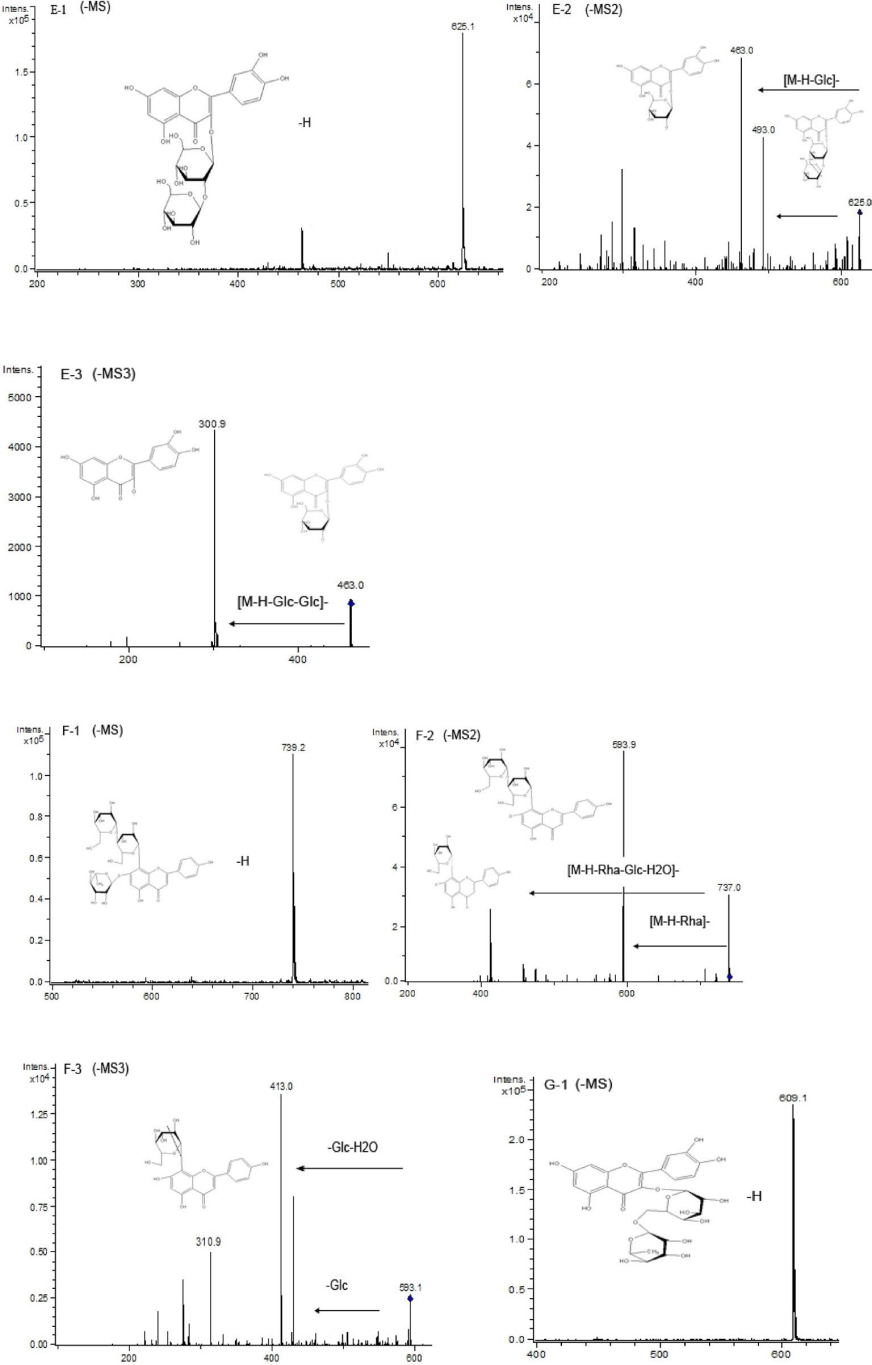
ESI (−) MS, MS^2^, and MS^3^ spectra of identified flavonoids in adzuki bean. Peak 5 (**E**): **E-1** MS spectrum of peak 5 ([M−H]^−^), **E-2** MS^2^ spectrum of the ion at *m/z* 625, **E-3** MS^3^ spectrum of the ion at *m/z* 463. Peak 6 (**F**): **F-1** MS spectrum of peak 6 ([M−H]^−^), **F-2** MS^2^ spectrum of the ion at *m/z* 739, **F-3** MS^3^ spectrum of the ion at *m/z* 593. Peak 7 (**G**): **G-1** MS spectrum of peak 7 ([M−H]^−^)

**Fig. 7:**
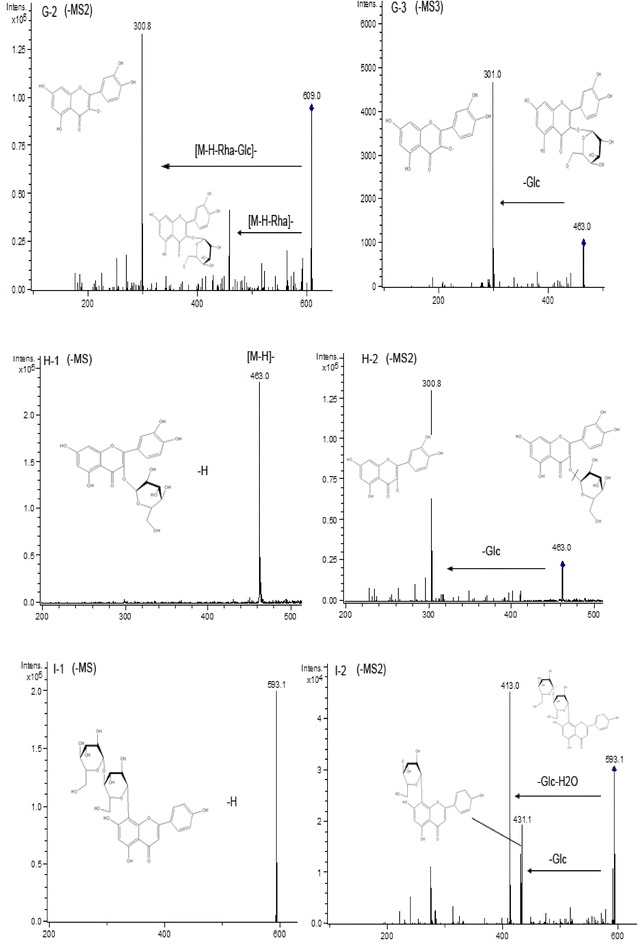
ESI (−) MS, MS^2^, and MS^3^ spectra of identified flavonoids in adzuki bean. Peak 7 (**G**): **G-2** MS^2^ spectrum of the ion at *m/z* 609, **G-3** MS^3^ spectrum of the ion at *m/z* 463. Peak 8 (**H**): **H-1** MS spectrum of peak 8 ([M−H]^−^), **H-2** MS^2^ spectrum of the ion at *m/z* 463. Peak 9 (**I**): **I-1** MS spectrum of peak 9 ([M−H]^−^), **I-2** MS^2^ spectrum of the ion at *m/z* 593

The peak 4 gave the retention time 25.7 min, which was different from the retention time of (+) catechin-*7*-*O*-*β*-*d*-glucopyranoside, and its parent ion [M−H]^−^ was at *m/z* 451 (Fig. 5D-1). As shown in Fig. 5D-2, the *m/z* 289 daughter ions were the main fragment ions of the parent ion at *m/z* 451. Comparing the HPLC–MS results of the standards of catechin to epicatechin, peak 4 was speculated to be epicatechin with one glucoside. According to the previous article [26], the peak 4 was speculated to be (+) epicatechin-*7*-*O*-*β*-*d*-glucopyranoside. For peak 5, the precursor ion *m/z* 625 with the retention time 27.9 min was observed in Fig. 6E-1. The CID of peak 5 produced main fragment ions at *m/z* 493, and *m/z* 463 in the ESI–MS^2^ and MS^3^ spectra (Fig. 6E-2, E-3). Hence, the peak 5 was speculated to be quercetin-*3*-*O*-glucoside-glucoside. The precursor ion of peak 6 was at *m/z* 739 (Fig. 6E-1), and one of the daughter ions was at *m/z* 593 of peak 6 (Fig. 6E-2), which was also the parent ion of peak 9 (Fig. 7I-1). Peak 6 lost an ion *m/z* 146, this suggested that the compound of peak 6 contained a rhamnose as compared to peak 9. In the ESI–MS^3^ spectrum (Fig. 6F-3), the fragmentation ion at *m/z* 431 produced from MS^2^ of the fragment ion at *m/z* 593. Peak 6 was speculated to be vitexin 4″-*O*-glucoside-rhamnose. Peaks 7 and 8 were respectively identified to be quercetin-*3*-*O*-rutinoside and quercetin-*3*-*O*-glucoside according to the retention times (37.5 min and 39.6 min, respectively) and MS information of their standards (Figs. 6, 7G, H).

For peak 9, the de-protonated molecular ion [M−H]− was at *m/z* 593(Fig. 7I-1), which molecular weight can be 594. In the ESI–MS^2^ spectrum (Fig. 7I-2), the fragment ions at *m/z* 431 and *m/z* 413 were the daughter ions from the precursor ion *m/z* 593. Based on the above information and the retention time of the standard vitexin and vitexin-*4″*-*O*-glucoside, peak 9 was finally confirmed to be vitexin-*4″*-*O*-glucoside. Similar to peak 6, the daughter ions *m/z* 431 and *m/z* 413 were also observed in the ESI–MS^2^ spectrum (Fig. 6F-2).

### Analysis of saponins in adzuki bean by HPLC–ESI–MS^n^

**Fig. 8:**
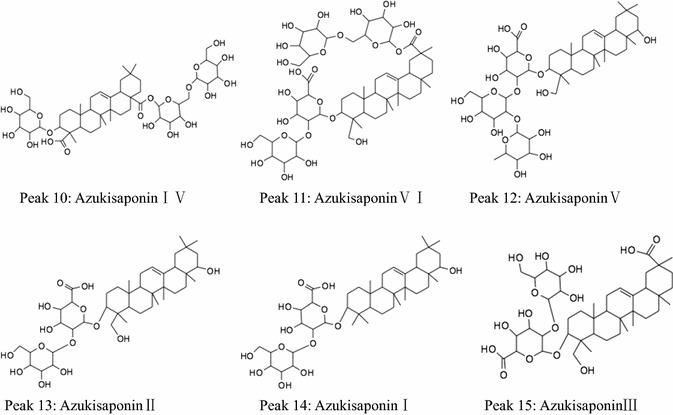
Chemical structures of saponins in adzuki bean identified by HPLC–ESI–MS^n^

**Fig. 9:**
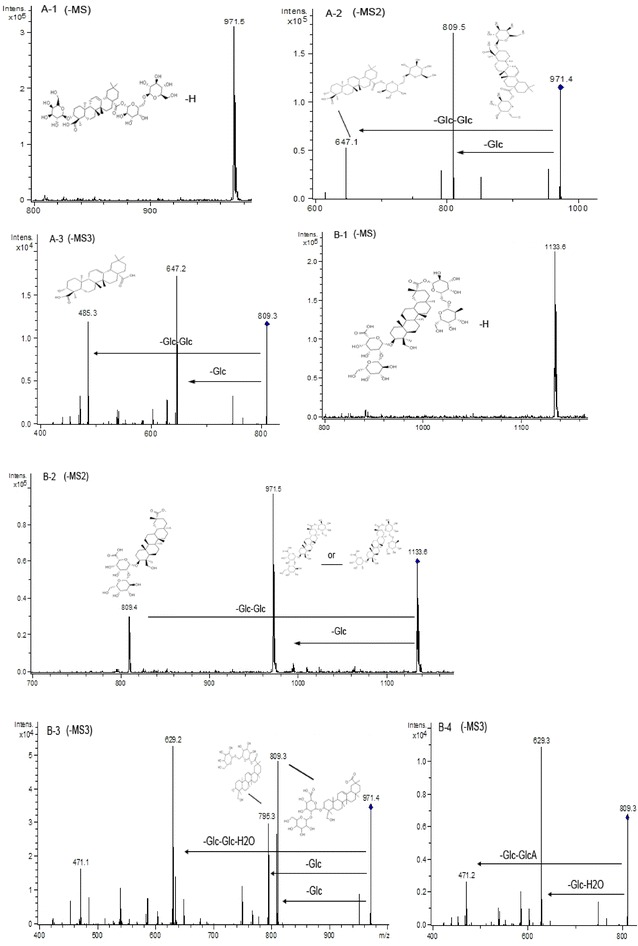
ESI (−) MS, MS^2^, and MS^3^ spectra of identified saponins in adzuki bean. Peak 10 (**A**): **A-1** MS spectrum of peak 10 ([M−H]^−^), **A-2** MS^2^ spectrum of the ion at *m/z* 971, **A-3** MS^3^ spectrum of the ion at *m/z* 809. Peak 11 (**B**): **B-1** MS spectrum of peak 11 ([M−H]^−^), **B-2** MS^2^ spectrum of the ion at *m/z* 1133, **B-3** MS^3^ spectrum of the ion at *m/z* 971; **B-4** MS^3^ spectrum of the ion at *m/z* 809

Peaks 10–16 (Fig. 3) were identified to be saponins of adzuki bean according to the retention times and MS information. Their structures were shown in Fig. 8. The following were the detailed analysis procedures. The molecular weight of peak 10 was confirmed to be 972 due to the existence of ion [M−H]^−^ at *m/z* 971 (Fig. 9A-1), and four fragment ions of MS^2^ at *m/z* 971 (809 [M−H-Glc]^−^, *m/z* 674 [M−H-Glc-Glc]^−^, *m/z* 629 [M−H-Glc-Glc-H_2_O]^−^, *m/z* 485 [M−H-Glc-Glc-Glc]^−^) (Fig. 9A-2), and the existences of fragment ions of MS^3^ at *m/z* 809 and *m/z* 647 (Fig. 9A-3), respectively. It was in tune with the standard and the previous article [27]. Taken together, peak 10 was identified as azukisaponin IV (Fig. 12).

**Fig. 10:**
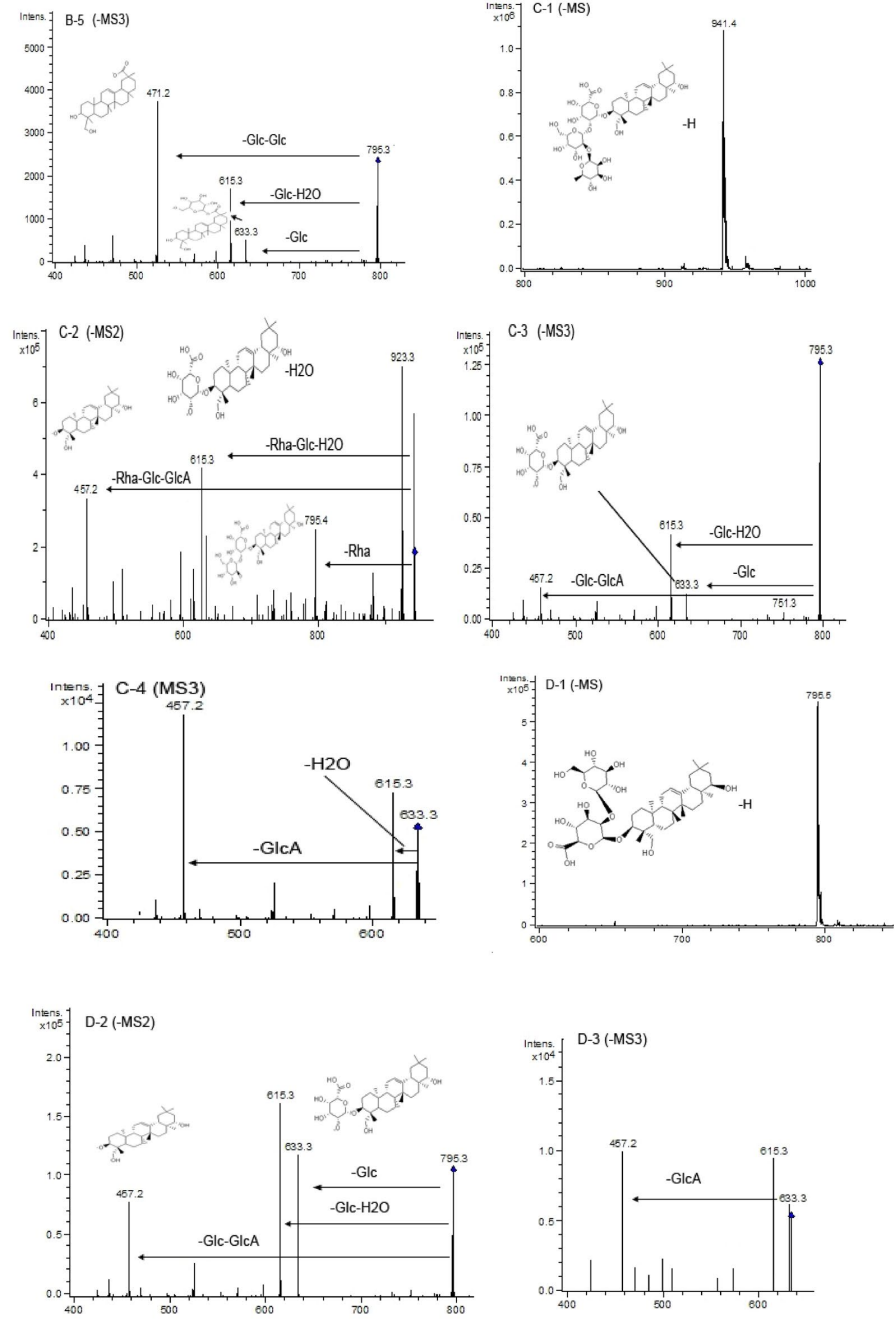
ESI (−) MS, MS^2^, and MS^3^ spectra of identified saponins in adzuki bean. Peak 11 (**B**): **B-5** MS^3^ spectrum of the ion at m/z 795. Peak 12 (**C**): **C-1** MS spectrum of peak 3 ([M−H]^−^), **C-2** MS^2^ spectrum of the ion at m/z 941, **C-3** MS^3^ spectrum of the ion at *m/z* 795; **C-4** MS^3^ spectrum of the ion at *m/z* 633. Peak 13 (**D**): **D-1** MS spectrum of peak 13 ([M−H]^−^), **D-2** MS^2^ spectrum of the ion at *m/z* 795, **D-3** MS^3^ spectrum of the ion at *m/z* 633

The CID of peak 11 with the [M−H]^−^ ion at *m/z* 1133 (Fig. 9B-1) resulted in fragments at *m/z* 971, *m/z* 809, *m/z* 795, and *m/z* 471 (Fig. 9B-2). MS^3^ spectrum at *m/z* 971 ([M−H-Glc]^−^), *m/z* 809 ([M−H-Glc-Glc]^−^) and *m/z* 795 ([M−H-Glc-Glc-Glc-H_2_O]^−^) were presented in Fig. 9B-3, B-4 and Fig. 10B-5, respectively. It was the same as the previous article [12]. So peak 11 was identified as azukisaponin VI.

The retention time of peak 12 was 63.2 min and the molecular ion was at *m/z* 941 (Fig. 10C-1). CID of the molecular ion of peak 12 produced three predominant fragments at *m/z* 795 ([M−H-Rha]^−^), *m/z* 633 ([M−H-Rha-Glc]^−^), and *m/z* 457 ([M−H-Rha-Glc]^−^) (Fig. 10C-2). Its MS^3^ spectrum at *m/z* 795 exhibited the fragment ions at *m/z* 633 and *m/z* 457, in which the ion *m/z* 795 lost a glucosyl, and a glucosyl with a glucuronic residue (Fig. 10C-3). MS^3^ spectrum at *m/z* 633 also produced the fragment ion *m/z* 457, which lost a glucuronic residue from the *m/z* 633 (Fig. 10C-4). It was identified to be azukisaponin V, which was consistent with the previous articles [24, 28, 29].

The retention time of peak 13 was 64.9 min, and the precursor ion was at *m/z* 795 (Fig. 10D-1), which suggested its molecular weight was 796. Its MS^2^ spectrum at *m/z* 795 and MS^3^ spectrum at *m/z* 633 were shown in Fig. 10D-2 and D-3, respectively. The identification of azukisaponin II of peak 13 was based on the above information and the similarity of MS^2^ with those reported by Kinjo et al. [24, 30].

**Fig. 11:**
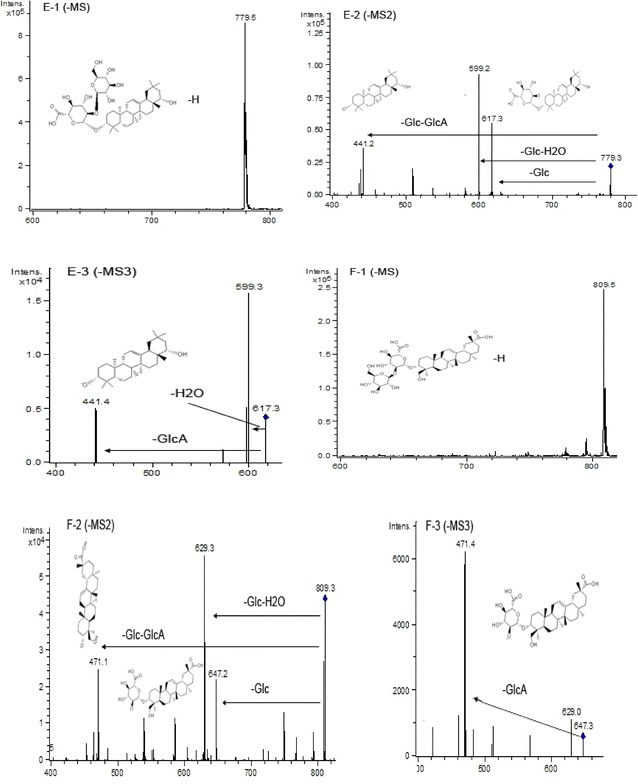
ESI (−) MS, MS^2^, and MS^3^ spectra of identified saponins in adzuki bean. Peak 14 (**E**): **E-1** MS spectrum of peak 14 ([M−H]^−^), **E-2** MS^2^ spectrum of the ion at *m/z* 779, **E-3** MS^3^ spectrum of the ion at *m/z* 617. Peak 15 (**F**): **F-1** MS spectrum of peak 15 ([M−H]^−^), **F-2** MS^2^ spectrum of the ion at *m/z* 809, **F-3** MS^3^ spectrum of the ion at *m/z* 647

Mass spectrometric analysis of peak 14 with the retention time at 66.4 min indicated that the molecular ion [M−H]^−^ present at *m/z* 779 (Fig. 11E-1). The fragment ions of *m/z* 617, *m/z* 599, and *m/z* 441 produced from the negative ESI-MS^2^ spectrum at *m/z* 779 ([M−H]^−^) in Fig. 11E-2. Its MS^3^ spectrum at *m/z* 617 further confirmed the result (Fig. 11E-3). Therefore, peak 14 was identified as azukisaponin I based on the above results and the previous article [12].

The molecular ion of peak 15 was at *m/z* 809 ([M−H]^−^) (Fig. 11F-1), and the molecular weight of peak 15 was 810. The fragment ion of *m/z* 647 indicated the loss of a glucose residue and *m/z* 471 indicated the losses of a glucose residue and a glucuronic residue. The detailed results were found in the MS^2^ spectrum at *m/z* 809 (Fig. 11F-2). In its MS^3^ spectrum, the main daughter ion at *m/z* 471 ([M−H-Glc-GlcA]^−^) was found from the fragment ion at *m/z* 647 (Fig. 11F-3). Moreover, it was consistent with the results reported by Kitagawa et al. [12]. Finally, peak 15 was identified as azukisaponin III.

**Fig. 12:**
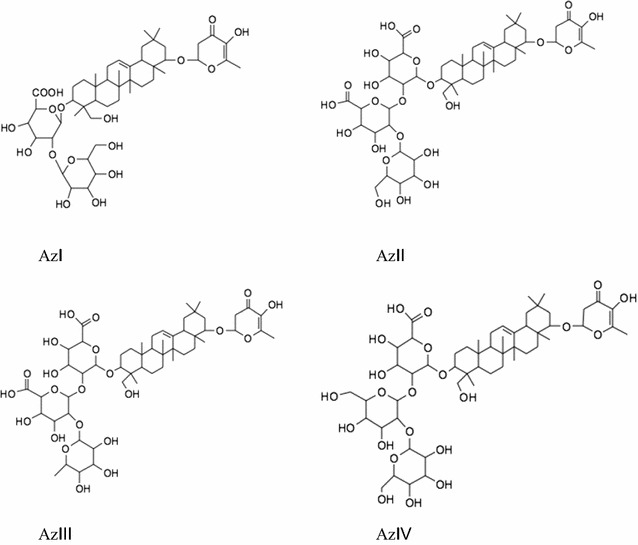
Chemical structures of Az saponins from adzuki bean (Adopted from Iida et al. [13, 14])

In the previous articles, other saponins were found in adzuki bean, namely Az I with the molecular weight of 922 [13], Az II with the molecular weight of 1098, Az III with the molecular weight of 1082, and Az IV with the molecular weight of 1084 [14]. The main differences of these saponins with the saponins of the present article were at the C-21 in Fig. 12. In the present article, no Az saponins were detected in adzuki bean samples. The reason may be their limited contents in adzuki bean or the procedure of preparation for adzuki bean samples.

### Quantification of flavonoids and saponins in adzuki bean

The program of time segments of MS analysis was employed to enhance sensitivity for flavonoids and saponins analysis. Among 15 compounds identified, four flavonoids (catechin, vitexin-4″-*O*-glucoside, quercetin-*3*-*O*-glucoside, and quercetin-*3*-*O*-rutinoside) and six saponins (azukisaponin I, II, III, IV, V, and VI) in adzuki bean samples were further quantified by external calibration using HPLC–MS methods with the program of “time segments” and extract ion chromatogram (EIC) analysis.

For making a standard curve of flavonoids, seven standard working solutions with 1, 2, 5, 20, 50 and 80, and 100 ng/mL were made by diluting from high concentration stock solutions, and analyzed by HPLC–DAD–MS^n^ using the above conditions, sequentially. Seven levels of saponins standard working solutions with 2, 5, 10, 40, and 60, 80, and 100 ng/mL were used to construct the standard curves. The curve of peak area (Y) versus flavonoid standard concentration (X) was plotted. The linear regression equation were Y = 42,769 X + 3 × 10^6^, (catechin, R^2^ = 0.994), Y = 2 × 10^6^ X + 5 × 10^6^, (quercetin-*3*-*O*-rutinoside, R^2^ = 0.9976), Y = 2 × 10^6^ X + 2 × 10^7^, (quercetin 3-glucoside, R^2^ = 0.9943), Y = 3 × 10^6^ X + 7 × 10^7^, (vitexin-4″-*O*-glucoside, R^2^ = 0.9969), Y = 2 × 10^6^ X + 4 × 10^7^, (azukisaponin IV, R^2^ = 0.9916), Y = 3 × 10^6^ X + 2 × 10^7^, (azukisaponin VI, R^2^ = 0.9906), Y = 2 × 10^6^ X + 3 × 10^7^, (azukisaponin V, R^2^ = 0.9985), Y = 4 × 10^6^ X + 2 × 10^7^, (azukisaponin II, R^2^ = 0.9923), Y = 1 × 10^6^ X + 4 × 10^7^, (azukisaponin I, R^2^ = 0.9911), and Y = 1 × 10^6^ X − 1 × 10^7^, (azukisaponin III, R^2^ = 0.9909), respectively.

The limit of detection (LOD) was calculated with Signal/Noise ratio better than 3, and the limit of quantification (LOQ) was calculated with Signal/Noise ratio better than 10. In the present article, the range for LOD of flavonoids standards was from 0.30 to 0.81 ng/mL, while the range for LOQ of saponins was from 0.7 to 1.42 ng/mL. The results showed that the contents of catechin (49.4 mg/g), quercetin-*3*-*O*-rutinoside (404.7 mg/g), quercetin-*3*-*O*-glucoside (90.1 mg/g) and vitexin-*4″*-*O*-glucoside (74.6 mg/g) in adzuki bean flavonoids extract were much higher than that of the adzuki bean total extract (12.4, 225.9, 21.4, and 36.7 mg/g, respectively). Meanwhile, the contents of azukisaponin IV (11.4 mg/g), azukisaponin VI (206.3 mg/g), azukisaponin V (283.2 mg/g), azukisaponin II (389.7 mg/g), azukisaponin I (5.4 mg/g), and azukisaponin III (27.6 mg/g) in adzuki bean saponins extract were much higher than that of adzuki bean total extract (6.6, 20.0, 165.9, 186.9, 8.9, and 79.0 mg/g, respectively) (Table 2).

**Table 2:** Flavonoids and saponins contents in extracts from Adzuki Bean

Peak no.	Compounds	Contents (mg/g ABTE)	Contents (mg/g ABF)	Contents (mg/g ABS)
2	Catechin	12.37	49.39	ND
7	Quercetin-*3*-*O*-rutinoside	225.99	404.73	ND
8	Quercetin-*3*-*O*-glucoside	21.37	90.08	ND
9	Vitexin-*4″*-*O*-glucoside	36.66	74.60	ND
10	Azukisaponin IV	6.63	ND	11.40
11	Azukisaponin VI	20.04	ND	206.35
12	Azukisaponin V	165.99	ND	283.21
13	Azukisaponin II	186.99	ND	389.73
14	Azukisaponin I	8.90	ND	5.42
15	Azukisaponin III	79.03	ND	27.58

*ABTE* adzuki bean total extract, *ABF* adzuki bean flavonoids, *ABS* adzuki bean saponins, *ND* not detected

## Conclusions

Flavonoids and saponins of adzuki bean have been produced by column chromatography and solvent precipitation. The present study has established a powerful method using HPLC–DAD–ESI–MS^n^ in electro spray negative mode to separate and characterize nine flavonoids and six saponins in adzuki bean rapidly. A simple and sensitive method has been established for quantification of flavonoids and saponins in adzuki bean samples. Current preparation and analysis of flavonoids and saponins from adzuki bean could promote pharmacological experiments and attain much more reasonable experimental results.
